# Plasmid stability is enhanced by higher-frequency pulses of positive selection

**DOI:** 10.1098/rspb.2017.2497

**Published:** 2018-01-10

**Authors:** Cagla Stevenson, James P. J. Hall, Michael A. Brockhurst, Ellie Harrison

**Affiliations:** 1 Department of Biology, University of York, York YO10 5DD, UK; 2 Department of Animal and Plant Sciences, University of Sheffield, Sheffield S10 2TN, UK

**Keywords:** experimental evolution, fluctuating selection, compensatory evolution, horizontal gene transfer, plasmid, mercury resistance

## Abstract

Plasmids accelerate bacterial adaptation by sharing ecologically important traits between lineages. However, explaining plasmid stability in bacterial populations is challenging owing to their associated costs. Previous theoretical and experimental studies suggest that pulsed positive selection may explain plasmid stability by favouring gene mobility and promoting compensatory evolution to ameliorate plasmid cost. Here we test how the frequency of pulsed positive selection affected the dynamics of a mercury-resistance plasmid, pQBR103, in experimental populations of *Pseudomonas fluorescens* SBW25. Plasmid dynamics varied according to the frequency of Hg^2+^ positive selection: in the absence of Hg^2+^ plasmids declined to low frequency, whereas pulses of Hg^2+^ selection allowed plasmids to sweep to high prevalence. Compensatory evolution to ameliorate the cost of plasmid carriage was widespread across the entire range of Hg^2+^ selection regimes, including both constant and pulsed Hg^2+^ selection. Consistent with theoretical predictions, gene mobility via conjugation appeared to play a greater role in promoting plasmid stability under low-frequency pulses of Hg^2+^ selection. However, upon removal of Hg^2+^ selection, plasmids which had evolved under low-frequency pulse selective regimes declined over time. Our findings suggest that temporally variable selection environments, such as those created during antibiotic treatments, may help to explain the stability of mobile plasmid-encoded resistance.

## 1. Introduction

Conjugative plasmids are extrachromosomal genetic elements that, alongside the genes required for their own replication, maintenance and transfer [1], carry cargos of accessory genes encoding functional traits. Common plasmid-encoded accessory traits include resistance to toxins, virulence factors and metabolic capabilities [2]. By transferring ecologically important functional traits within and between bacterial lineages and species, plasmids can accelerate bacterial adaptation [3]. Therefore, the dynamics and stability of conjugative plasmids in bacterial populations have potentially important implications for understanding bacterial evolution [4,5]. Nevertheless, it remains challenging to explain the long-term stability of plasmids. This is because plasmid maintenance is frequently costly for the bacterial host cell [6]. Although such costs may be outweighed by the benefits of plasmid-encoded functional traits in some environments [7], theory predicts that plasmids should be evolutionarily unstable whether parasitic (i.e. costs outweigh benefits) or mutualistic (i.e. benefits outweigh costs) [8–11]. In the short term, parasitic plasmids are expected to decline in frequency owing to negative selection, because observed rates of horizontal transmission appear too low to counteract this process [10,12]. While mutualistic plasmids can be temporarily favoured by positive selection for accessory gene functions, they are expected to decline in frequency over longer evolutionary timescales. This is because the useful accessory genes can be integrated into the chromosome, rendering the plasmid-backbone dispensable. Thus, consistent positive selection for accessory genes should favour plasmid-free cells with the accessory traits on their chromosome, which outcompete plasmid-bearers who still pay the cost of plasmid carriage [10,13,14].

In both natural and clinical environments, plasmids are likely to experience temporally variable selection, resulting in fluctuating positive selection for the accessory genes they carry [15–17]. Recent theory suggests that temporally heterogeneous environments where plasmids experience pulsed positive selection may favour their maintenance through two non-mutually exclusive mechanisms [13,18]. Firstly, rare pulses of strong positive selection can theoretically promote the maintenance of conjugative plasmids carrying accessory gene functions. This occurs because plasmid-free cells outcompete both plasmid-bearers and cells with chromosomal accessory genes between bouts of positive selection, but only the plasmid-encoded copies of the accessory genes can conjugate into these plasmid-free cells. These plasmid-bearing transconjugant cells can then sweep to high frequency upon the next pulse of positive selection [13]. By contrast, where pulses of positive selection are frequent, the frequency of plasmid-free cells and thus the benefits of conjugation are reduced. Therefore, under constant or high-frequency pulses of positive selection, cells with chromosomal accessory genes are favoured at the expense of accessory genes encoded on the conjugative plasmid. Secondly, pulses of positive selection have been shown to promote compensatory evolution to ameliorate the cost of plasmid carriage thereby weakening negative selection against the plasmid-backbone. This occurs because positive selection temporarily increases the population size of plasmid-bearing cells thus increasing the probability that they will gain compensatory mutations [18]. Compensatory evolution appears to be a fairly general mechanism by which plasmid survival is ensured, it has been observed in a range of bacteria–plasmid interactions [18–20] and across environments where the fitness effect of plasmid acquisition ranges from parasitic to mutualistic [19].

Here, we tested how the frequency of pulsed positive selection affected plasmid stability (i.e. the stable maintenance of the plasmid in the bacterial population). We experimentally evolved populations of *Pseudomonas fluorescens* SBW25 with the mercury-resistance (Hg^R^) plasmid pQBR103 [19] across a range of treatments varying in the frequency of exposure to toxic concentrations of mercuric ions (Hg^2+^). Mercuric ions are normally lethal to the bacterial cell, binding to protein sulfhydryl groups and causing major cellular disruption [21]. However, in this bacteria–plasmid system, pQBR103 encodes a Tn5042 transposon which in turn harbours a mercury-resistance operon, *mer*, that catalyses reduction of Hg^2+^ to a less toxic form Hg^0^. Thus, while in the absence of Hg^2+^, pQBR103 imposes a large fitness cost on SBW25, at higher Hg^2+^ concentrations this fitness cost is offset by benefit of Hg^R^ [7,19]. Populations were propagated under one of six treatments: in the absence of mercury, under constant mercury selection or pulsed mercury selection at varying time intervals (i.e. every 2, 4, 8 or 16 transfers). After 16 transfers of these selection regimes, all populations were propagated for a further 16 transfers in the absence of Hg^2+^ to test the effect of prior evolution under the varying frequencies of pulsed positive selection on longer-term plasmid stability. Throughout the experiment, we tracked plasmid prevalence and the frequency of phenotypes associated with a previously described mechanism of compensatory evolution in this bacteria–plasmid interaction.

## 2. Material and methods

### (a) Strains and culture conditions

Experiments used *P. fluorescens* SBW25 [22] differentially marked with either gentamicin resistance (Gm^R^) or streptomycin resistance + lacZ (Sm^R^lacZ) cassettes [7,23] allowing them to be distinguished on selective agar plates as previously described [7,19]. pQBR103 was conjugated into the Gm^R^ background using standard methods [19,24]. All experiments were conducted in 6 ml King's B (KB) broth in 30 ml microcosms shaking at 180 r.p.m. and incubated at 28°C. The carrying capacity of KB microcosms is approximately 1 × 10^10^ colony forming units ml^−1^; electronic supplementary material, figure S1.

### (b) Selection experiment

Independent overnight cultures of plasmid-bearing, mercury resistant (Hg^R^) and plasmid-free, mercury sensitive (Hg^S^) strains were mixed at a 1 : 1 ratio and 60 µl (approx. 10^9^ cells ml^−1^) were used to inoculate treatment microcosms. Six replicate populations were established for each mercury treatment. Populations were propagated by 1% serial transfer every 48 h for a total of 32 transfers. Two ‘constant' treatments were established with either 0 or 40 µM HgCl_2_ added at each transfer. In the four pulsed treatments, populations were grown without mercury except for 40 µM HgCl_2_ added every 2, 4, 8 or 16 transfers. After 16 transfers addition of HgCl_2_ was stopped and all populations were propagated in 0 µM HgCl_2_ for a further 16 transfers to measure plasmid stability in the absence of selection. Every two transfers population densities of each marker background were determined by diluting and plating onto KB agar supplemented with 50 µg ml^−1^ X-gal and 5% powdered milk solution. In addition, frequency of the Hg^R^ phenotype was determined by selective plating onto KB agar supplemented with 40 µM HgCl_2_ and 50 µg ml^−1^ X-gal and 5% milk. The addition of milk powder allowed us to determine the frequency of *gacA/gacS* mutants (Gac^−^) in the populations. Previously, it was shown that loss of function mutation to the *gacA/gacS* bacterial regulatory system is the main mechanism of compensatory evolution in this system ameliorating the cost of pQBR103 carriage in *P. fluorescens* SBW25 [19]. The gacA/gacS system positively regulates expression of an extracellular protease allowing colonies of wild-type Gac^+^ SBW25 to digest a halo zone of clearing around the colony on milk plates [25], allowing Gac^+^ phenotypes to be easily distinguished from Gac^−^ mutants, which cannot form the halo. The frequency of transconjugants was determined by scoring Sm^R^lacZ marked cells that grew on Hg^2+^ plates, forming a blue colony. To check that Hg^R^ colonies were unlikely to have arisen by mutation, we quantified the frequency of spontaneous Hg^R^ mutations against 40 µM Hg^2+^, using the fluctuation test assay protocol described in [26]. We never detected any spontaneous Hg^R^ mutants, strongly suggesting mercury resistance requires the *mer* operon and could not have evolved *de novo* in our experiments.

At the end of the experiment 24 Hg^R^ clones from each population were isolated and colony polymerase chain reaction (PCR) was used to test whether the plasmid was still present or whether it was lost following chromosomal acquisition of the resistance genes. PCRs targeted *oriV* (for: 5′-TGCCTAATCGTGTGTAATGTC-3′ and rev: 5′-ACTCTGGCCTGCAAGTTTC-3′) to determine the presence of the plasmid-backbone and *merA* (for: 5′-TGAAAGACACCCCCTATTGGAC-3′) and rev: 3′-TTCGGCGACCAGCTTGATGAAC-3′) to determine the presence of the *mer* operon.

### (c) Statistical analysis

All analyses were conducted in R statistical package v. 3.1.3 [27]. Packages used were ‘nlme' and ‘userfriendlyscience'. For all analyses of Hg^R^ plasmid dynamics the mercury-free treatment was removed so that mercury treatments were compared to one another. Comparisons across the mercury pulsed treatments looking at average prevalence of Hg^R^, average proportion of transconjugants, proportion of Gac^−^ phenotypes at *T*_16_, time to first Gac^−^ mutant and average Gac^−^ frequency over time were analysed using Welch's ANOVA with mercury treatment as a fixed effect to adjust for non-homogeneous variance across treatments. Comparisons of Gac^−^ dynamics across plasmid-bearing and plasmid-free populations were analysed using Welch's ANOVA with the presence of plasmid as a fixed effect. Maintenance of Hg^R^ over time between *T*_16_ and *T*_32_ was analysed using linear mixed effects models with mercury treatment and time as fixed effects, and random effects of population on intercept and slope to account for repeated sampling of populations through time. Fixed effects were assessed using likelihood ratio tests on nested models.

## 3. Results

### (a) Hg^R^ plasmid dynamics varied between mercury treatments

**Figure 1.**
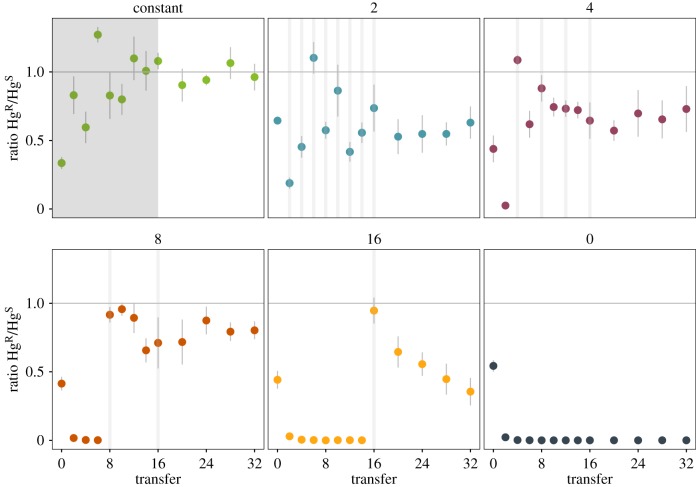
Pulses of mercury selection maintain pQBR103. The proportion of Hg^R^ (ratio of Hg^R^ counts over Hg^S^ counts) was determined over time across the six selection treatments (constant mercury, mercury pulsed every 2, 4, 8 and 16, and absence of mercury). Grey bars indicate transfers where mercury was applied. Points represent means ± standard errors of six replicate populations. Colours represent each pulsed mercury treatment.

Populations were propagated for 16 transfers either without mercury, with mercury addition every transfer (constant mercury) or in pulsed treatments where mercury exposure occurred at varying time intervals (i.e. every 2, 4, 8 or 16 transfers), and the frequency of Hg^R^ was measured every second transfer. In all treatments where Hg^R^ was detected, PCR analysis on endpoint clones revealed that Hg^R^ remained associated with the plasmid (i.e. we did not detect any mutants which had acquired chromosomal *mer* and lost the plasmid-backbone). In the mercury-free treatment, Hg^R^ cells harbouring pQBR103 were rapidly outcompeted by plasmid-free Hg^S^ cells, as expected based on the known fitness cost associated with carrying pQBR103 [19] (figure 1). By contrast, under constant mercury selection Hg^R^ was maintained at high prevalence in all populations. During the first 16 transfers Hg^R^ prevalence varied across pulsed treatments, such that mean prevalence averaged over time was significantly higher under more frequent pulses (electronic supplementary material, figure S2; effect of mercury treatment: *F*_4,25_ = 55.77, *p* < 0.001).

In all pulsed mercury treatments, plasmid prevalence declined prior to the initial mercury pulse. However, in all cases, a single mercury pulse was sufficient to sweep Hg^R^ to high frequencies, such that by transfer 16, by which time every pulsed treatment had experienced at least 1 mercury pulse, Hg^R^ was at high frequency in all populations and did not differ significantly between pulsed treatments (effect of mercury treatment: *F*_4,25_ = 1.77, *p* = 0.166). The increase in Hg^R^ frequency was particularly striking in populations from the treatment with the lowest frequency of mercury pulse (i.e. single pulse at *T*_16_) where, prior to the pulse, Hg^R^ was virtually undetectable (figure 1). Together these results demonstrate across the first 16 transfers, that higher-frequency pulses of positive selection favoured high plasmid prevalence and also that even rare positive selection events could boost plasmid persistence, at least in the short term.

### (b) Compensatory evolution occurred across all mercury treatments

**Figure 2.**
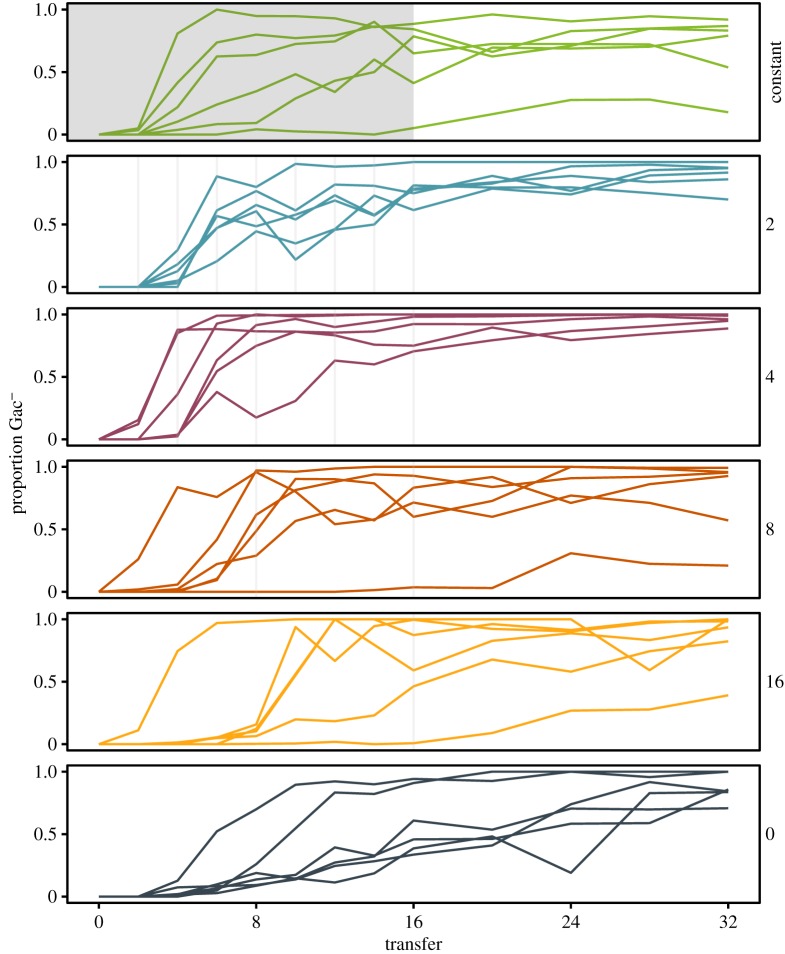
Gac mutations sweep through all Hg^R^ populations regardless of selective regime. The proportion of Gac^−^ phenotypes within the Hg^R^ population was determined over time across the six selection treatments (constant mercury, mercury pulsed every 2, 4, 8 and 16, and absence of mercury). Grey bars indicate transfers where mercury was applied. Lines represent the six replicate populations. Colours represent each pulsed mercury treatment.

We screened the Hg^R^ fraction of each population to determine the presence of phenotypes associated with compensatory evolution. In this bacteria–plasmid interaction, we have previously described a mechanism of compensatory evolution associated with the loss of function in the bacterial *gacA/gacS* two-component regulator [19]. The *gacA/gacS* system is encoded by the bacterial chromosome and controls the expression of genes involved in a broad range of biological functions including secondary metabolism, virulence and motility [25,28]. Addition of milk powder to agar plates allowed us to screen for Gac^−^ phenotypes: cells carrying *gacA/gacS* compensatory mutations were unable to produce the extracellular proteases capable of digesting milk. We, therefore, used this phenotype to compare the frequency of Gac^−^ phenotypes between treatments. Gac^−^ phenotypes arose in both plasmid-bearing and plasmid-free cells (shown in figure 2 and electronic supplementary material, figure S3, respectively). This is not necessarily surprising given that *gacA/gacS* loci are known to have an elevated mutation rate relative to the genome as a whole [29]. Among the plasmid-bearers we found that Gac^−^ phenotypes appeared rapidly in all mercury treatments and were maintained for the duration of the experiment (figure 2). This was not observed in plasmid-free control populations (electronic supplementary material, figure S3), where Gac^−^ phenotypes appeared later (plasmid-bearing versus plasmid-free: *F*_1,10_ = 62.8, *p* < 0.001), and remained at significantly lower frequency (plasmid-bearing versus plasmid-free: *F*_1,10_ = 17.06, *p* = 0.002). This is consistent with our previous data showing that deletion of *gacA/gacS* was only beneficial in cells with the pQBR103 plasmid, but had no significant fitness effects in plasmid-free SBW25 [19]. Within plasmid-containing treatments there was no significant effect of mercury treatment on Gac^−^ frequency in the plasmid-bearing population over the selective period of the experiment (i.e. averaged over transfer 1–16) (effect of mercury treatment: *F*_5,30_ = 1.99, *p* = 0.108) or the proportion Gac^−^ mutants at *T*_16_ (effect of mercury treatment: *F*_4,25_ = 0.99, *p* = 0.433), suggesting that amelioration of the plasmid cost was strongly favoured across all conditions regardless of mercury exposure [19]. Furthermore, there was no significant effect of mercury treatment on time taken for Gac^−^ mutants to arise: Gac^−^ phenotypes arose rapidly across all the plasmid-bearing populations (effect of mercury: *F*_5,30_ = 0.74, *p* = 0.598).

### (c) Infrequent pulses promoted plasmid transfer into Hg^S^ recipients

**Figure 3.**
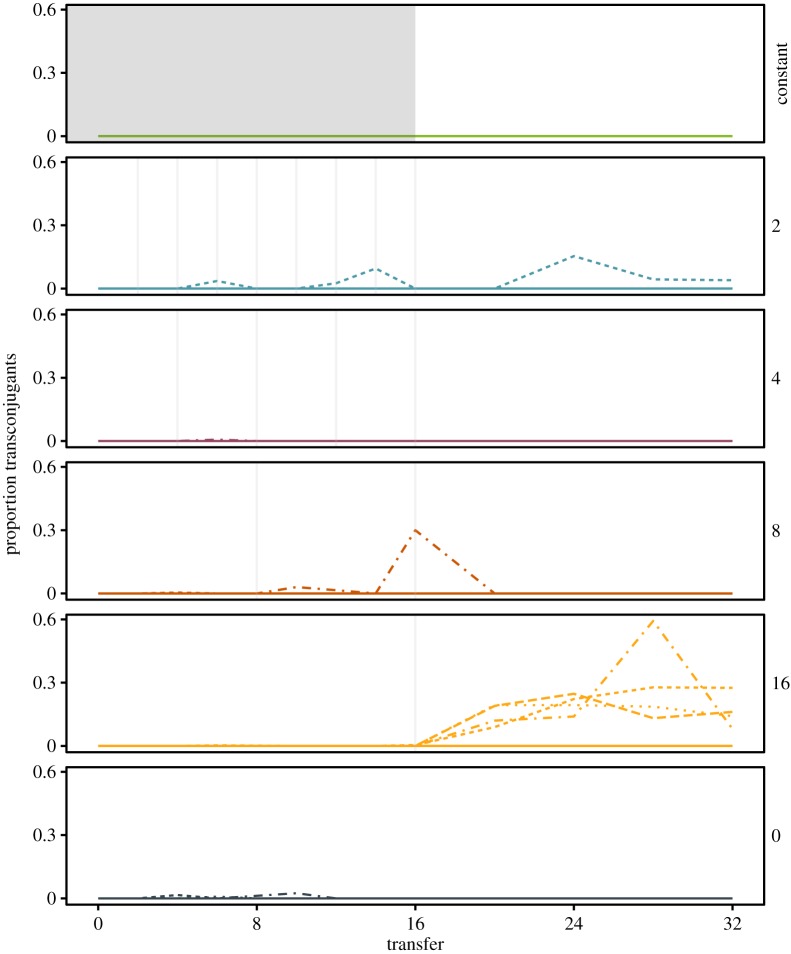
Infrequent pulses promote plasmid transfer into Hg^S^ recipients. The proportion of transconjugants within the Hg^R^ population was determined over time across the six selection treatments (constant mercury, mercury pulsed every 2, 4, 8 and 16, and absence of mercury). Grey bars indicate transfers where mercury was applied. Lines represent the six replicate populations. Colours represent each pulsed mercury treatment.

Theory suggests that longer intervals between pulses of selection may favour conjugative plasmid transfer [13]. This occurs by allowing the survival and propagation of plasmid-free Hg^S^ bacteria which can then act as recipient hosts for the plasmid [30]. The frequency of transconjugants across each population revealed that the level of conjugative plasmid transfer significantly increased with decreasing frequency of pulsed mercury selection (figure 3; data for individual replicate populations shown in the electronic supplementary material, figure S5; effect of mercury treatment: *F*_4,25_ = 7.19, *p* = 0.001). This is likely to have been driven by frequent mercury pulses reducing the frequency of plasmid-free cells (electronic supplementary material, figure S1), whereas less frequent mercury pulses allowed plasmid-free cells to rise to high frequency, allowing greater opportunity for conjugation from the remaining plasmid-bearing cells. Therefore, in treatments with rare pulses of positive selection, conjugation indeed appears to play a larger role in the persistence of Hg^R^ within populations.

### (d) High-frequency pulses stabilized Hg^R^ plasmids over the longer term

After *T*_16_, all populations were propagated without mercury, to test how adaptation to the various selection regimes had affected plasmid stability in the absence of positive selection. Hg^R^ stability varied according to the past frequency of pulsed positive selection (figure 1; time × mercury treatment: 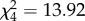, *p* = 0.0076). Comparisons revealed that this effect was largely driven by the populations subjected to a single mercury pulse at *T*_16_ (*b* = −0.0327, *t*_114_ = −2.63, *p* = 0.0096), where Hg^R^ steadily declined over time in the absence of mercury selection, whereas Hg^R^ was stable in populations from all the other pulsed mercury treatments.

## 4. Discussion

Understanding the conditions that favour the stability of conjugative plasmids is important for understanding bacterial evolution [8,10,11,13,31]. Most experimental studies of plasmid stability have used constant environmental conditions, yet in nature, bacteria inhabit environments that are likely to be temporally variable with pulses of positive selection for plasmid-borne traits [16,17,32]. While there have been theoretical studies of the impact of pulsed positive selection on conjugative plasmid stability [13], there have been few experimental tests (however, see [18] and [33] for studies on non-conjugative plasmids and integrases, respectively). Here, we show short- and longer-term effects of the frequency of pulsed positive selection on the stability of a mercury-resistance plasmid. In the short term, constant or frequent pulses of positive selection allowed plasmids to be maintained at higher prevalence, but even in treatments where the plasmid had declined to undetectable levels, the first pulse of positive selection was sufficient to sweep the plasmid to high prevalence. Surprisingly, the high plasmid prevalence observed under frequent pulses did not appear to affect the rate of compensatory evolution via loss of function mutations to the *gacA/gacS* pathway [19], which arose in all mercury environments. In the longer term, however, plasmids that only experienced a single pulse of positive selection did appear to be at a disadvantage: following the removal of positive selection, plasmids evolved under high frequency or constant positive selection remained at high prevalence, whereas plasmids evolved under the lowest frequency of positive selection declined.

Previous theoretical analysis of plasmid stability predicted that horizontally transferable, plasmid-encoded resistance would be favoured over chromosomally encoded resistance by rare pulses of strong positive selection [13]. This is predicted to occur because plasmid-free cells, which pay no cost of carrying the resistance gene, can outcompete both plasmid-encoded and chromosomally encoded resistant genotypes in the intervals between pulses of positive selection. While this leads to the loss of chromosomal resistance, plasmid-encoded resistance can transfer by conjugation into the population of plasmid-free cells, and these transconjugants may then sweep to high frequency following the next pulse of positive selection [13]. Although we did not observe the emergence of chromosomally encoded resistance in our study, even though this outcome is possible in our experimental system [19], we did observe the out-competition of plasmid-bearers by plasmid-free cells during long intervals between infrequent pulses of positive selection. Moreover, consistent with the prediction of the model [13], under the lowest frequency of pulsed positive selection we observed a significantly higher proportion of transconjugant cells during the experiment, suggesting that conjugation played a more important role in the persistence of the plasmid where positive selection was rarest. This is consistent with previous work which demonstrated that conjugation played a larger role in the maintenance of the Hg^R^ plasmid pQBR57 in the absence, rather than presence, of positive mercury selection [30]. The balance of vertical versus horizontal transmission of genes determines population genomic structure and thus the evolutionary potential of populations to changing environmental conditions [34]. As plasmids can spread to a wide range of hosts [35], our finding that infrequent pulses of positive selection favoured horizontal transfer via conjugation suggests that we may expect to observe functional genes in a broader range of bacterial species when positive selection is a rare event [30,36].

Contrary to our prediction, based on recent theory and experimental data [18], we did not observe higher rates of compensatory evolution (via loss of function mutation to the *gacA/gacS* pathway) under higher-frequency pulsed positive selection even though such environments did support higher plasmid prevalence. By contrast, we observed that compensatory phenotypes evolved rapidly and rose to high frequency among plasmid-bearers across all our mercury environments. Compensatory evolution in this bacteria–plasmid interaction is associated with loss of function in the *gacA/gacS* two-component regulatory system [19], which activates the expression of a wide range of secondary metabolism and secreted products [25,28]. Consistent with our findings here, it was previously found that Gac^−^ mutants arose in parallel across a wide range of mercury concentrations, suggesting that neither the strength nor the frequency of positive selection has a major effect on the process of compensatory evolution in this system [19]. A potential explanation for this widespread prevalence of compensatory evolution across the range of positive selective environments, is that *gacA/gacS* appear to be contingency loci in *P. fluorescens* [37], i.e. loci with an elevated mutation rate relative to the rest of the genome [29]. Consequently, the abundant supply of compensatory mutations in this system may obscure any effect of the frequency or strength of positive selection. It is likely that loss of *gacA/gacS* function may be detrimental in more complex, natural environments, where the suite of genes activated within the gac regulon perform important functions, notably associated with host colonization and interspecific competition including the production of toxins and antibiotics [25,28]. Under such conditions, where expression of the Gac regulon is advantageous, the bacteria–plasmid assemblage would be forced to find alternative mechanisms of amelioration, and the frequency of pulsed positive selection may have a stronger effect on the rate of compensatory evolution.

Interestingly, we observed contrasting longer-term effects of the history of positive selection on the fate of plasmids following removal of positive selection. Unlike plasmids evolved under high-frequency pulses of positive selection, plasmids evolved under the lowest frequency of pulsed positive selection declined in prevalence in mercury-free environments. This cannot be explained by a lack of compensatory evolution (via loss of function mutation to the *gacA/gacS* pathway), because we observed compensatory phenotypes at high frequency among plasmid-bearers in all mercury selection environments. At present we do not know the evolutionary mechanism driving this effect. However, one possibility is that where plasmids have very recently swept from very low (in some cases undetectable) frequency, these lineages may be poorly adapted compared with the plasmid-free cells. This could arise because, until the recent pulse of mercury selection, the plasmid-free lineage had been at far higher population density than the plasmid-bearers and therefore had access to a higher mutational supply allowing them greater opportunity to adapt to the abiotic environment [38].

Pulsed positive selection is likely to be a common feature of both environmental contamination and clinical antibiotic treatments, such that positive selection for plasmid-encoded traits is likely to be temporally heterogeneous [15–17]. Our findings suggest that this is likely to have both short- and longer-term effects on plasmid stability. High-frequency pulsed positive selection increases plasmid prevalence and promotes the longer-term survival of plasmids in bacterial populations in the absence of positive selection, whereas low-frequency pulsed positive selection increases the importance of horizontal gene transfer and may lead to plasmid-encoded functional genes spreading into, and subsequently being selected in, a greater diversity of bacterial hosts. Crucially, we show how even very rare periods of positive selection can be sufficient to sweep plasmids from undetectable levels to high frequency. Thus plasmids need not be present at high frequency to have an impact on bacterial evolution in temporally heterogeneous environments, because even vanishingly rare plasmids can enhance the responsiveness of bacterial populations to changing and uncertain conditions [39].

## Supplementary Material

Figure S1

## Supplementary Material

Figure S2

## Supplementary Material

Figure S3

## Supplementary Material

Figure S4

## Supplementary Material

Figure S5

## Data accessibility

Data deposited to Dryad (http://dx.doi.org/10.5061/dryad.1cd62) [40].
