# Supplementary figures and images for: Plasmid stability is enhanced by higher-frequency pulses of positive selection
Source: Proc Biol Sci. 2018 Jan 10;285(1870):20172497. doi: 10.1098/rspb.2017.2497 (PMC5784203; doi:10.1098/rspb.2017.2497)

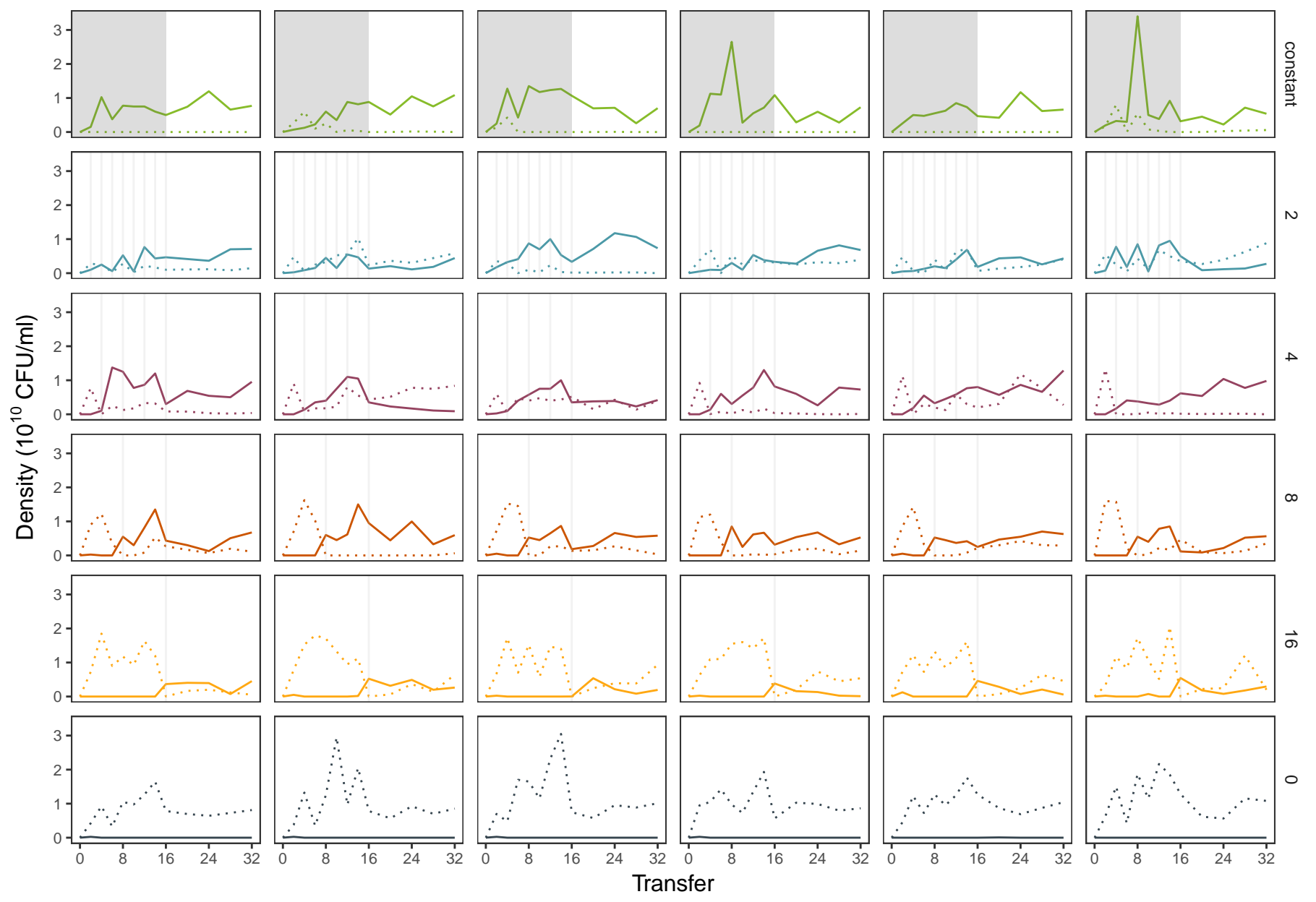

Supplement: Figure S1 [file rspb20172497supp1.pdf]

Average proportion  $Hg^R$  during mercury selection

constant

2

4

8

16

Frequency of mercury selection

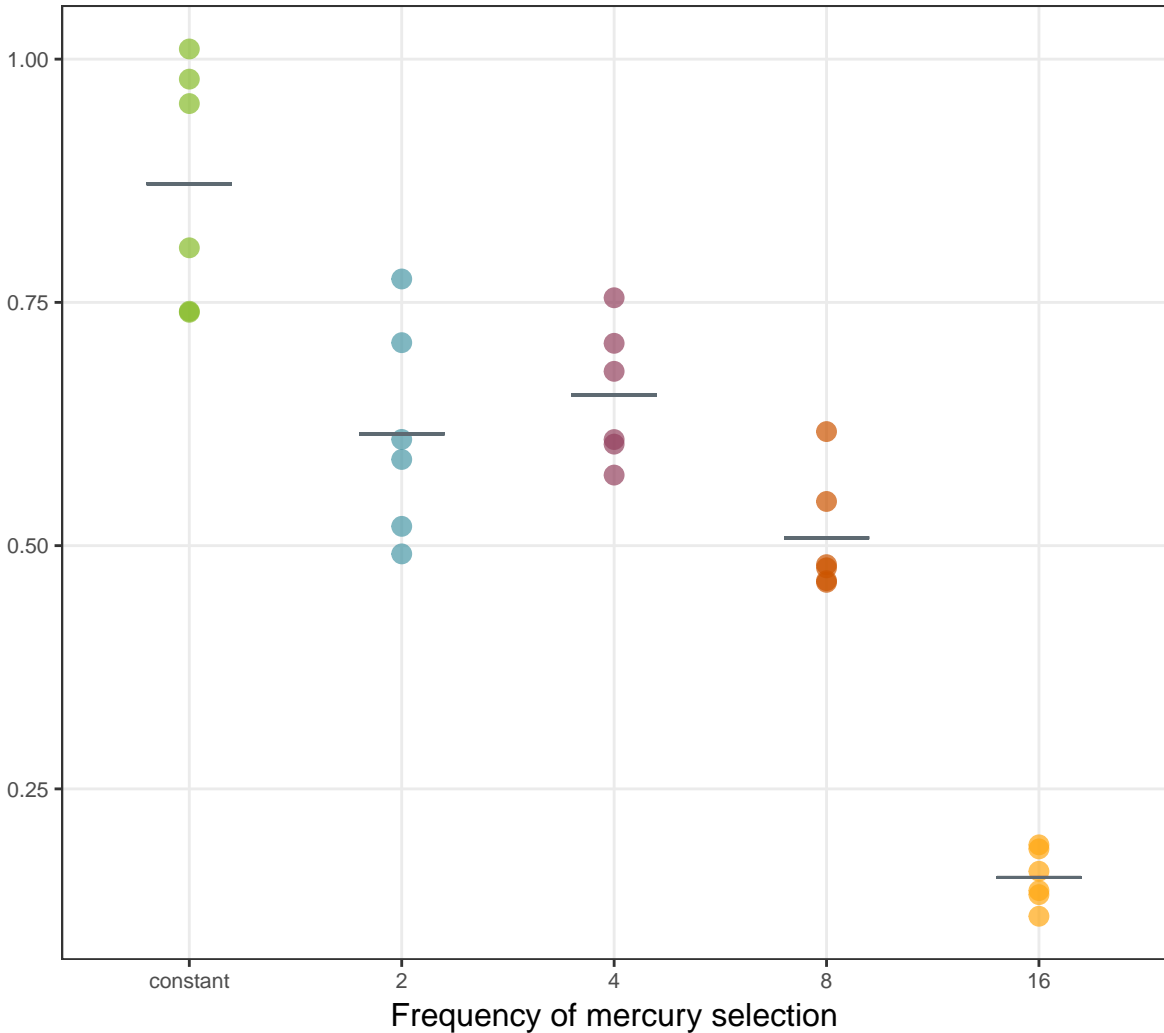

Supplement: Figure S2 [file rspb20172497supp2.pdf]

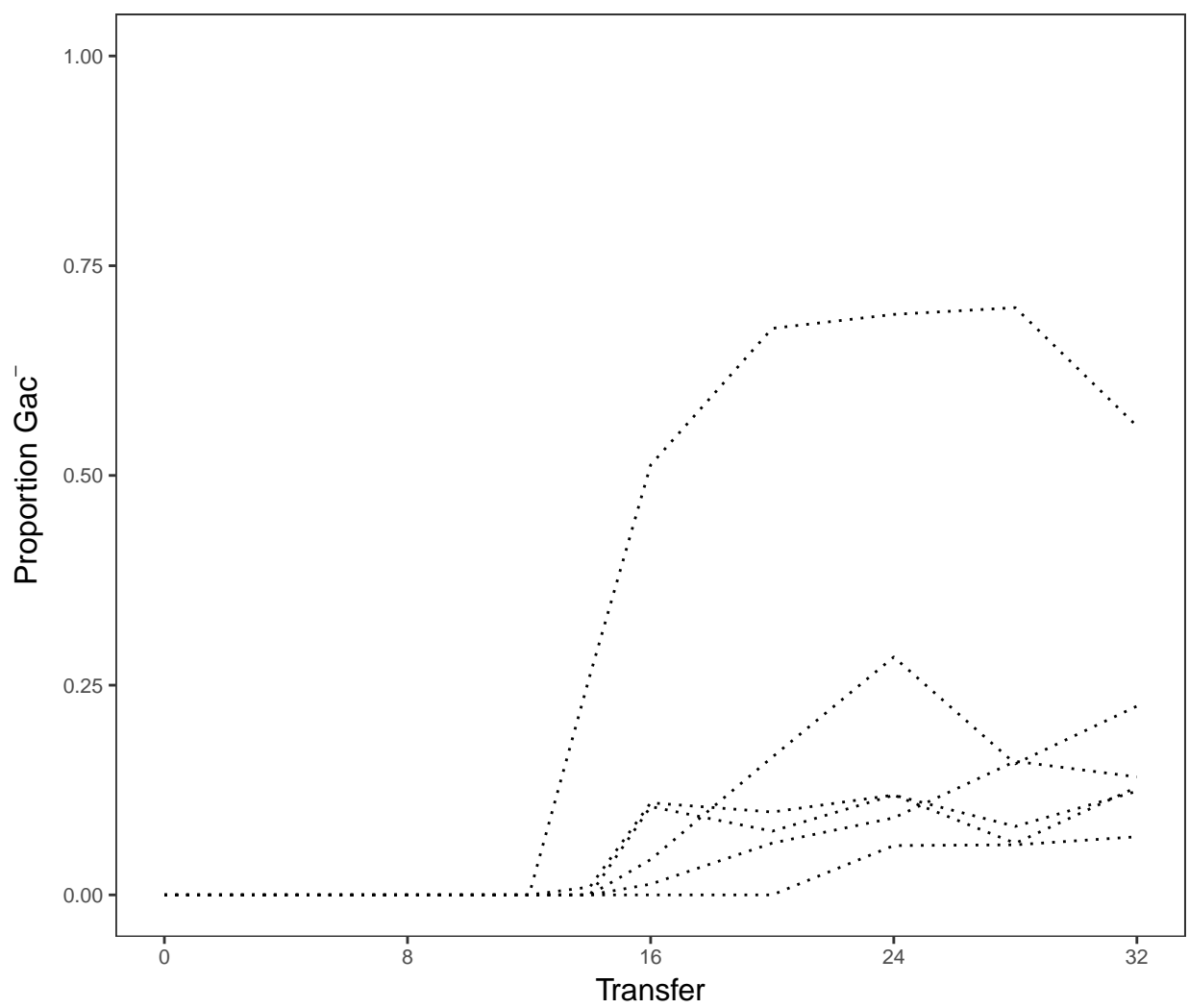

Supplement: Figure S3 [file rspb20172497supp3.pdf]

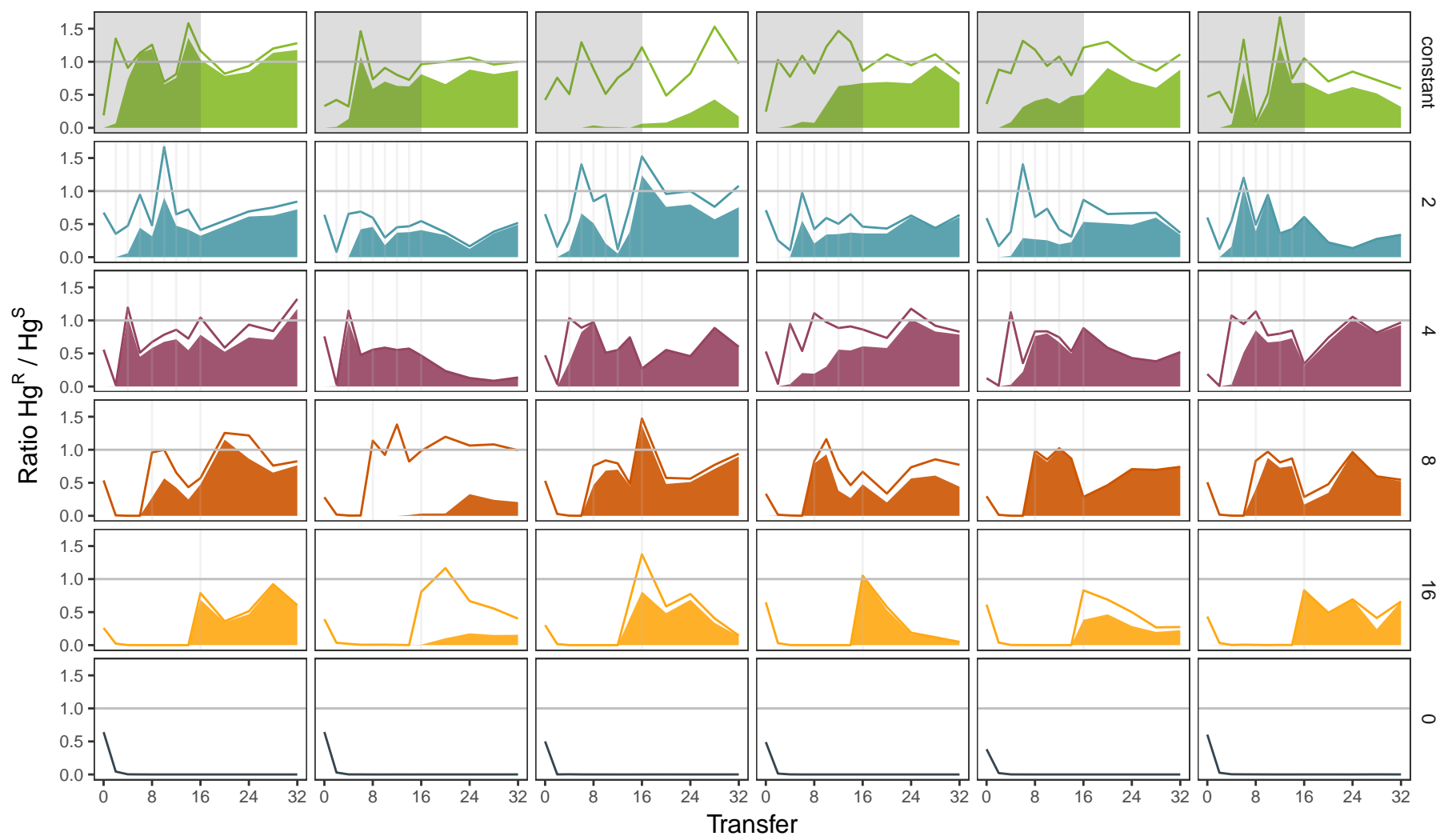

Supplement: Figure S4 [file rspb20172497supp4.pdf]

Density ( $10^{10}$  CFU/ml)

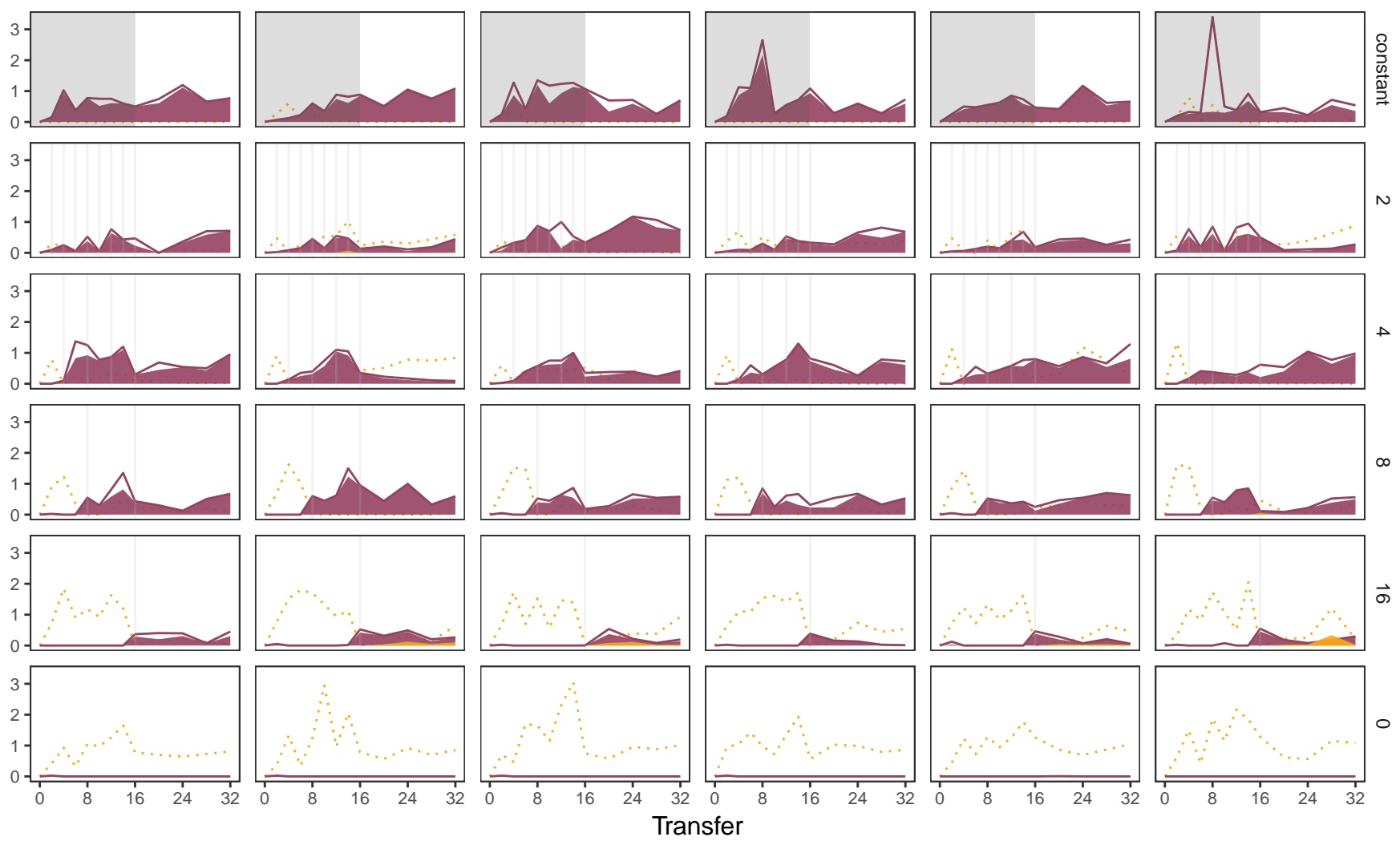

Supplement: Figure S5 [file rspb20172497supp5.pdf]
